# Non-neuronal cholinergic system contributes to corticosteroid resistance in chronic obstructive pulmonary disease patients

**DOI:** 10.1186/s12931-016-0467-8

**Published:** 2016-11-08

**Authors:** Javier Milara, Angela Cervera, Alfredo de Diego, Celia Sanz, Gustavo Juan, Amadeu Gavaldà, Montserrat Miralpeix, Esteban Morcillo, Julio Cortijo

**Affiliations:** 1 Department of pharmacology, faculty of medicine, Jaume I University, Castellón, Spain; 2 Pharmacy Unit, University General Hospital Consortium, Valencia, Spain; 3 CIBERES, Health Institute Carlos III, Valencia, Spain; 4 Respiratory Unit, University General Hospital Consortium, Valencia, Spain; 5 Respiratory Unit, University and Polytechnic La Fe Hospital, Valencia, Spain; 6 Department of Pharmacology, Faculty of Medicine, University of Valencia, Valencia, Spain; 7 Almirall, R&D Centre, Barcelona, Spain; 8 Health Research Institute INCLIVA, Valencia, Spain; 9 Research and teaching Unit, University General Hospital Consortium, Valencia, Spain; 10 Unidad de Investigación Clínica, Consorcio Hospital General Universitario, Avenida tres cruces s/n, E-46014 Valencia, Spain

**Keywords:** Neutrophils, COPD, Corticosteroid resistance, Aclidinium bromide, Non-neuronal cholinergic system

## Abstract

**Background:**

Inhaled corticosteroid (ICS) with long-acting beta-2 agonists is a well-documented combination therapy for chronic obstructive pulmonary disease (COPD) based on its additive anti-inflammatory properties. By contrast, the recommendation of ICS in combination with long-acting muscarinic antagonist (LAMA) is not evidence-based. In this study, neutrophils obtained from COPD patients were used to compare the anti-inflammatory effects of aclidinium bromide (a long-acting muscarinic antagonist) with corticosteroids and their potential additive effect.

**Methods:**

Human sputum and blood neutrophils were isolated from healthy individuals (*n* = 37), patients with stable COPD (*n* = 52) and those with exacerbated COPD (*n* = 16). The cells were incubated with corticosteroid fluticasone propionate (0.1 nM–1 μM), aclidinium bromide (0.1 nM–1 μM) or a combination thereof and stimulated with 1 μg of lipopolysaccharide/ml or 5 % cigarette smoke extract. Levels of the pro-inflammatory mediators interleukin-8, matrix metalloproteinase-9, CCL-5, granulocyte-macrophage colony-stimulating factor and interleukin-1β were measured and the mechanisms of corticosteroid resistance evaluated at the end of the incubation.

**Results:**

The non-neuronal cholinergic system was over-expressed in neutrophils from COPD patients, as evidenced by increases in the expression of muscarinic receptors (M2, M4 and M5), choline acetyltransferase and vesicular acetylcholine transporter. Aclidinium bromide demonstrated anti-inflammatory effects on neutrophils from COPD patients, reversing their resistance to corticosteroids. Additive effects of combined aclidinium bromide and fluticasone propionate in blocking M2 receptor levels, inhibiting phosphoinositide 3-kinase-δ and enhancing the glucocorticoid response element transcription factor were demonstrated and were accompanied by an increase in the corticosteroid-induced expression of anti-inflammatory-related genes.

**Conclusions:**

LAMAs potentiate the anti-inflammatory effects of corticosteroids in neutrophils from COPD patients in vitro, thus providing a scientific rationale for their use in combination with corticosteroids in the treatment of COPD.

**Electronic supplementary material:**

The online version of this article (doi:10.1186/s12931-016-0467-8) contains supplementary material, which is available to authorized users.

## Background

Chronic obstructive pulmonary disease (COPD) is characterised by airway and systemic inflammation leading to airway remodelling and obstruction that are not completely reversible. The current first-line maintenance treatment for COPD involves the use of bronchodilators, including long-acting muscarinic antagonists (LAMAs) and long-acting beta agonists (LABAs). Inhaled corticosteroids (ICS), despite their good activity in asthma, are much less effective in improving lung function and have little or no effect in controlling the underlying chronic inflammation in COPD [1]. Combined therapies, consisting of ICS + LABAs, LABA + LAMA, as well as LAMA monotherapy are common options for COPD patients at increased risk of an exacerbation of moderate symptoms [2–4]. Triple therapy based on ICS in combination with LABAs and LAMAs is indicated in patients with severe COPD who are at risk of disease exacerbation [5]. In the latter patients, the effective agents include the phosphodiesterase 4 (PDE4) inhibitor roflumilast, an anti-inflammatory agent used to reduce exacerbations of chronic bronchitis [6].

The rationale for drug combinations to achieve additive or synergistic effects in the treatment of COPD comes from clinical randomised studies and can be explained by pharmacologic molecular interactions. Thus, for example, LABAs increase corticosteroid responsiveness by reversing glucocorticoid receptor alpha (GRα) phosphorylation at serine 226 (Ser226) and promoting GRα nuclear translocation [7, 8]. Recent evidence indicates that LABAs reverse corticosteroid insensitivity by attenuating oxidative stress and inhibiting phosphoinositide-3-kinase delta (PI3Kδ), thus allowing the efficient action of histone deacetylase-2 (HDAC2) and GRα [9]. These mechanisms, which are shared with theophylline [7] and, as shown recently, roflumilast [10], explain the clinical benefits of combination therapy to improve corticosteroid anti-inflammatory effects in COPD. However, the use of LAMAs + ICS is thus far not evidence-based, although its potential efficacy is suggested by the current Global Initiative for Chronic Obstructive Lung Disease (GOLD 2015) guidelines.

COPD is associated with increased pulmonary vagal activity [11]. Muscarinic antagonists are effective drugs for the treatment of COPD because their anticholinergic effect results in the relaxation of airway smooth muscle [12]. In addition, a non-neuronal cholinergic system has been demonstrated in human airway epithelial cells [13], lung fibroblasts [14], alveolar macrophages [15] and sputum neutrophils [16] and represents a previously unappreciated regulatory pathway in pulmonary inflammation and remodelling. Because dysfunction of the non-neuronal cholinergic system appears to be involved in the pathophysiology of COPD [17], the potential anti-inflammatory and anti-remodelling effects of muscarinic antagonists, shown in preclinical models [18], may be of added value to their established bronchodilation in the management of chronic respiratory diseases. The persistent activation of neutrophils, as the primary effector cell involved in the inflammatory process of COPD, contributes to its pathogenesis [19]. Current anti-inflammatory therapies based on corticosteroids poorly regulate neutrophil activation, which has limited their clinical effectiveness [10, 20]. The aim of this study was to provide scientific evidence of the anti-inflammatory effects of the anticholinergic agent aclidinium bromide in the corticosteroid-insensitive neutrophils from COPD patients. Our results provide support for the scientific rationale regarding the use of combined LAMAs and ICS therapy in patients with COPD.

## Methods

All reagents were obtained from Sigma-Aldrich (St. Louis, MO, USA) unless otherwise stated.

### Patients

Sputum neutrophils, peripheral blood neutrophils and whole blood were obtained from COPD patients who were smokers and from healthy non-smoking controls. The study population consisted of 52 patients with stable COPD, defined according to the 2013 GOLD guidelines, 16 patients with severe exacerbated COPD and 37 age-matched non-smoking healthy controls with normal lung function. Neutrophils from patients with stable COPD were used in the mechanistic experiments, and those from patients with exacerbated COPD to measure the basal expression of non-neuronal cholinergic components in neutrophils. All patients with exacerbated disease were hospitalised because of airway bacterial infections, confirmed by bacteriological analysis of blood and sputum. Cell samples from patients with exacerbated COPD were collected before starting treatment with oral corticosteroids. The minimum washout period in stable COPD patients for sampling sputum or blood was 4 days, which avoided effects of chronic medication on the results. All COPD patients were current smokers and had bronchitis. In the patients with stable COPD, there were no disease exacerbations within 2 weeks prior to sample collection. Routine lung function tests were performed to evaluate forced vital capacity (FVC), forced expiratory volume in one second (FEV1) and FEV1/FVC ratio using a Vitalograph® αIII spirometer (Vitalograph, Maids Moreton, UK). The clinical features of the study population are summarised in Table 1. This project was approved by the local Ethics Committee of the University General Hospital of Valencia, Spain. Written informed consent was obtained from each patient or volunteer before starting sputum/blood sampling and lung function testing.

**Table 1:** Clinical features. COPD: chronic obstructive pulmonary disease; FEV1: forced expiratory volume in one second; FVC: forced vital capacity; Pack-yr = 1 year smoking 20 cigarettes-day. Data are mean ± SD. *P < 0.05 related to Healthy subjects

	Healthy (*n* = 37)	Stable COPD (*n* = 52)	Exacerbated COPD (*n* = 16)
Age, yr	66.1 ± 6	65.1 ± 14	63.8 ± 8.4
Sex (M/F)	27/10	35/17	12/4
Tobacco consumption, pack-yr	0	35.2 ± 6*	42.3 ± 13*
FEV1, % pred	98 ± 3	53.2 ± 3*	38.0 ± 13*
FVC, % pred	96 ± 4	90.2 ± 6	89.5 ± 8
FEV1/FVC %	98 ± 3	50.1 ± 6*	46.2 ± 9*
GOLD 1 (mild) patients, no.	0	0	0
GOLD 2 (moderate) patients, no.	0	36	3
GOLD 3 (severe) patients, no.	0	16	10
GOLD 4 (very severe) patients, no.	0	0	3
Receiving inhaled steroids, no.	0	26	16
Receiving theophyllines, no.	0	0	0
Receiving long-acting b2-agonist, no.	0	49	16
Receiving anticholinergics, no.	0	41	13
Total peripheral blood neutrophils	4.2 ± 0.3 x 10^9^/L	8.2 ± 1.3 x 10^9^/L*	9.9 ± 0.2 x 10^9^/L*

### Human neutrophil isolation

Neutrophils were isolated from peripheral venous blood and cultured as previously outlined [21], using 3 % dextran 500 (in 0.9 % saline) together with Ficoll-Paque Histopaque 1077 (Amersham Pharmacia Biotech, Barcelona, Spain) at a ratio of 2:1. The neutrophil preparations were >97 % pure as assessed by Giemsa staining and had viability of >99 % as measured by trypan blue exclusion. Neutrophils from spontaneous sputum (~2 ml) were collected from patients with stable and exacerbated COPD and processed with dithiothreitol using established methods [22]. Sputum cell pellets were resuspended in RPMI 1640 supplemented with 10 % foetal calf serum, 1 % penicillin-streptomycin and 1 mmol l-glutamine/L at a concentration of 1 × 10^6^ cells/ml. An aliquot containing 4 × 10^5^ cells was incubated on a 24-well plate for 1 h at 37 °C in humidified 5 % CO_2_. Preparations containing < 95 % neutrophils were discarded. Neither the purity nor the viability of the cell preparations was affected by the different experimental conditions of the study.

### Preparation of cigarette smoke extract solutions

Cigarette smoke extract (CSE) was prepared as previously outlined [23]. Briefly, the smoke of a research cigarette (2R4F; Tobacco Health Research, University of Kentucky, Lexington, KY, USA) was generated by a respiratory pump (Rodent Respirator 680; Harvard Apparatus, March-Hugstetten, Germany) through a puffing mechanism mimicking the human smoking pattern (3 puffs/min; 1 puff 35 ml; each puff of 2 s duration, 0.5 cm above the filter) and was bubbled into a flask containing 25 ml of pre-warmed (37 °C) RPMI 1640 culture medium. The resulting CSE solution was considered as 100 % CSE and used for experiments within 30 min of preparation. CSE 10 % corresponded approximately to the exposure associated with smoking two packs of cigarettes per day [24]. To test for cytotoxicity/apoptosis induced by CSE, isolated neutrophils were treated with CSE concentrations of up to 5 % for 6 h. No significant difference in the lactate dehydrogenase level (lactate dehydrogenase cytotoxicity assay; Cayman Chemical, Madrid, Spain) or annexin V-FITC was observed between the CSE and control groups (data not shown).

### Cell stimulations and cytokine assays

Sputum and peripheral blood neutrophils were adjusted to 500 × 10^3^ cells per well in 24-well plates and incubated in RPMI 1640 for 1 h at 37 °C, 5 % CO_2_. The cells were then left untreated or treated with long-acting muscarinic antagonist aclidinium bromide (0.1 nM–1 μM; Almirall Laboratories, Barcelona, Spain), muscarinic antagonist atropine (0.1 nM–1 μM), corticosteroid fluticasone propionate (0.1 nM–1 μM), long-acting beta 2 agonist formoterol (0.01 nM–100 nM), long-acting beta 2 agonist salmeterol (0.1 nM–1 μM), the muscarinic receptor type 3 (M3) inhibitor p-fluoro-hexahydrosiladifenidol (pFHHSid; 10 nM, 1 μM), the M2 inhibitor methoctramine (100 nM, 1 μM), the PI3K inhibitor LY294002 (1 μM) or the nicotinic receptor antagonist hexamethonium (100 μM) for 1 h before they were stimulated with 1 μg of lipopolysaccharide (LPS)/ml), CSE 5 % or 10 μM carbachol. In other experiments, 10 U of acetylcholinesterase (ACheE)/ml was added 1 h before the stimulus to remove extracellular acetylcholine and during the 6-h period of LPS or CSE stimulation. CSE 5 % was selected as a stimulus in sputum neutrophils because LPS alone did not increase interleukin (IL)-8 levels over basal values, as previously reported [25].

The stimuli and drugs were incubated together with the cells for 6 h. Supernatants were collected and centrifuged at 120 × g for 5 min. The cell-free supernatant was used to measure IL-8, metalloproteinase-9 (MMP9), CCL-5, granulocyte-macrophage colony-stimulating factor (GM-CSF) and IL-1β. Cellular extracts were used to measure mRNA expression after 6 h of cell stimulation. IL-8 levels were measured using a commercially available enzyme-linked immunosorbent assay kit for IL-8 (R&D Systems, Nottingham, UK) according to the manufacturer’s protocol. MMP9, CCL-5, GM-CSF and IL-1β were measured using LUMINEX technology, in accordance with the manufacturer’s protocol.

### Real-time RT-PCR and siRNA experiments

Total RNA was isolated from sputum or peripheral blood neutrophils using the TriPure® isolation reagent (Roche, Indianapolis, IN, USA). The integrity of the extracted RNA was confirmed with Bioanalyzer (Agilent, Palo Alto, CA, USA). Reverse transcription was performed using 300 ng of total RNA with a TaqMan reverse transcription kit (Applied Biosystems, Perkin-Elmer Corporation, CA, USA). cDNA was amplified using specific primers together with probes predesigned by Applied Biosystems for organic transporter cation 1 (OTC1; cat. no. Hs00222691_m1), OTC2 (cat. no. Hs01010726_m1), OTC3 (cat. no. Hs00427552_m1), choline acetyltransferase (ChAT; cat. no. Hs00252848_m1), high-affinity choline transporter (ChT1; cat. no. Hs00222367_m1), M1 (cat. no. Hs00265195_s1), M2 (cat. no. Hs00265208_s1), M3 (cat. no. Hs00265216_s1), M4 (cat. no. Hs00265219_s1), M5 (cat. no. Hs00255278_s1), β2 adrenergic receptor (β2ADR; cat. no. Hs00240532_s1), vesicular acetylcholine transporter (cat. no. VAChT; Hs00268179_s1), macrophage migration inhibitory factor (MIF; cat. no. Hs00236988), mitogen-activated protein kinase phosphatase 1 (MKP-1; cat. no. Hs00610256), PI3K-δ (cat. no. Hs00192399), HDAC2 (cat. no. Hs00231032), GRα (cat. no. Hs00353740_m1), cysteine-rich secretory protein LCCL domain-containing 2 (CRISPLD2; cat. no. Hs00230322_m1) and glucocorticoid-induced leucine zipper (GILZ; cat. no. Hs00608272_m1) genes in a 7900HT Fast Real-Time PCR system (Applied Biosystems) using Universal Master Mix (Applied Biosystems).

Expression of the target gene was reported as the fold increase or decrease relative to the expression of GAPDH as an endogenous control (Applied Biosystems; 4310884E). The mean value of the replicates for each sample was calculated and expressed as the cycle threshold (Ct). The level of gene expression was then calculated as the difference (ΔCt) between the Ct value of the target gene and the Ct value of GAPDH. The fold changes in the target gene mRNA levels were expressed as 2^−ΔCt^.

Small interfering RNA (siRNA), including the scrambled siRNA control, was purchased from Ambion (Huntingdon, Cambridge, UK). Cultured human bronchial epithelial cells Beas2B were transfected with 50 nM of a commercial siRNA against the M2 (PN 4392421; Ambion, Austin TX, USA) or M3 (PN 4390815; Ambion, Austin TX, USA) gene or with 50 nM of the siRNA control (Ambion, Huntingdon, Cambridge, UK) in serum-free and antibiotic-free medium. After 6 h, the medium was aspirated and replaced with medium containing serum for a further 48 h. Lipofectamine-2000 (Invitrogen, Paisley, UK), at a final concentration of 2 μg/mL, was used as the transfection reagent.

### Glucocorticoid response element transfection assay

Beas2B epithelial cells were seeded (40,000 cells/well) and cultured for 24 h under a 5 % CO_2_/air atmosphere at 37 °C in 96-well plates containing Dulbecco’s modified Eagle’s medium (DMEM). The Cignal GRE reporter assay kit (Qiagen, cat. no. 336841) was used to monitor the activity of glucocorticoid receptor-induced signal transduction pathways in cultured cells, following the manufacturer’s indications. First, the cells were transfected with M2- or M3-gene-targeted siRNA or the scrambled siRNA control as described above. After 24 h, the cells were transfected with Cignal reporter (100 ng), the Cignal negative control (100 ng) and the Cignal positive control (100 ng) in Opti-MEM serum-free culture medium using Lipofectamine 2000 (Invitrogen) as the transfection reagent. Subsequently, the cells were incubated with the transfection reagents at 37 °C in a 5 % CO_2_ incubator for 16 h and then pre-incubated for another 6 h with different fluticasone propionate and drug combinations at different concentrations in DMEM.

After the second 6-h incubation, the luciferase assay was developed using the Dual-Luciferase Reporter Assay system (Promega, cat. no. 1910) following the manufacturer’s protocol. In brief, the growth medium was removed from the cultured cells, which were then washed gently with phosphate-buffered saline (PBS). After complete removal of the rinse solution, passive lysis buffer 1× was added. The culture plate was then placed on an orbital shaker for gentle shaking at room temperature for 15 min. The luciferase assay reagent II (LAR II) was prepared by resuspending the provided lyophilised luciferase assay substrate in 10 mL of the supplied luciferase assay buffer II. One hundred microliters of LAR II was predispensed into the appropriate number of wells of a white 96-well plate, followed by 20 μl of cell lysate and mixing by pipetting two or three times. The assay plate was placed in a luminometer (Victor Luminometer, Perkin-Elmer, Madrid, Spain) and firefly luciferase activity was measured. Just before use, the Stop & Glo reagent was prepared by diluting 1 volume of the Stop & Glo substrate with 50 volumes of Stop & Glo buffer. After the measurement of luciferase activity, 100 μl of Stop & Glo reagent was dispensed in the corresponding wells and a second luminometer reading was initiated, recording *Renilla* luciferase activity. The data are expressed as 2× the GRE-reporter fold induction of luciferase relative to that of unstimulated cells.

### Western blot

Western blot analysis was used to detect changes in p-ERK1/2, p-p38, MKP1 and phospho-serine 226-GR. Neutrophils incubated in RPMI 1640 were treated with fluticasone propionate, aclidinium bromide or a combination thereof for 1 h and stimulated with LPS for 30 min. The cells were then centrifuged and total protein was extracted as previously outlined [26].

Electrophoresis was carried out using 20 μg of protein (denatured) and a molecular mass protein marker (Bio-Rad Kaleidoscope marker; Bio-Rad Laboratories Ltd.) loaded onto an acrylamide gel consisting of a 5 % acrylamide stacking gel and a 10 % acrylamide resolving gel. After electrophoresis at 100 V for 1 h, the proteins were transferred from the gel to a polyvinylidene difluoride membrane using a wet blotting method. The membrane was blocked with 5 % Marvel in PBS containing 0.1 % Tween20 (PBS-T), probed with a rabbit anti-human p-ERK1/2 (1:1000) antibody (monoclonal antibody; Cell Signaling, Boston, MA, USA; cat. no. 4376S) and normalised to total rabbit anti-human ERK1/2 (1:1000) antibody (monoclonal antibody; Cell Signaling; cat. no. 4695); rabbit anti-human phospho-p38 (1:1000) antibody (monoclonal antibody; Cell Signaling; cat. no. 4631) normalised to total rabbit anti-human p38 (1:1000) antibody (monoclonal antibody; Cell Signaling; cat. no. 9212); rabbit anti-human polyclonal MKP1 (1:1000) antibody (Assay Biotech; cat. no. B1099) normalised to total mouse anti-human β-actin (1:10,000) antibody (monoclonal antibody; cat. no. A1978; Sigma); or rabbit anti-human polyclonal phospho-GR-Ser226 (1:1000) antibody (Novus Biologicals, Littleton, CO, USA; cat. no. NB100-92540), rabbit anti-human polyclonal M1 (1:1000) antibody (Sigma; cat. no. M9808), rabbit anti-human polyclonal M2 (1:1000) antibody (Sigma; cat. no. M9558), rabbit anti-human polyclonal M3 (1:1000) antibody (Sigma; cat. no. M0194), mouse anti-human monoclonal M4 (1:1000) antibody (Merck Millipore, Madrid, Spain; cat no. MAB1576), or rabbit anti-human polyclonal M5 (1:1000) antibody (Novus Biologicals; cat. no. NBP1-00907) normalised to mouse anti-human monoclonal GRα (1;1000) antibody (BD Biosciences, Franklin Lakes, NJ, USA; cat. no. 611227). The enhanced chemiluminescence method of protein detection (ECL Plus; Amersham GE Healthcare, Little Chalfont, UK) was used to detect labelled proteins. Protein expression was quantified by densitometry relative to normalised antibody expression using the software GeneSnap version 6.08. The results are expressed as ratios of the endogenous controls as appropriate.

### PI3Kδ activity

To measure PI3Kδ activity, neutrophils from COPD patients were isolated and then incubated with aclidinium bromide (10 nM), atropine (100 nM), LY294002 (1 μM), methoctramine (1 μM) or pFHHSid (1 μM) for 1 h. The cells were stimulated with LPS for 30 min and then centrifuged. Total protein was extracted and the amount measured using the Bio-Rad assay (Bio-Rad Laboratories Ltd., Hemel Hempstead, UK) to ensure equal amounts (500 μg) in the immunoprecipitation reaction with anti-PI3-kinase δ antibody (p110δ; ab32401; Abcam, Cambridge, UK). PI3K activity was measured using the PI3-kinase activity ELISA (cat. no. k-1000s; Echelon Bioscience, Salt Lake City, UT, USA), in accordance with the manufacturer’s protocol. In brief, PI3-K reactions were run with the class I PI3-K physiological substrate PI [4, 5] P2 (PIP2). The enzyme reactions, PIP3 standards and controls were then mixed and incubated with PIP3 binding protein, which is highly specific and sensitive to PIP3. This mixture was transferred to a PIP3-coated microplate for competitive binding and the amount of PIP3 produced by PI3-K was then detected, using a peroxidase-linked secondary detector and colourimetric detection, comparing the enzyme reactions with a PIP3 standard curve. The results are expressed as pmol PI [3–5] P_3_ per mg of protein.

### Data analysis

The data were subjected to a parametric analysis, with *p* < 0.05 considered indicative of statistical significance. Parametric data are expressed as the mean ± SD of n experiments using a Student’s *t*-test and one-way or two-way analysis of variance (ANOVA) followed by a Bonferroni post hoc test. The concentration of aclidinium bromide, fluticasone propionate, formoterol or salbutamol producing 50 % inhibition (IC_50_) was calculated from the concentration-response curves by nonlinear regression in neutrophils from healthy individuals and COPD patients.

## Results

### Basal activation of non-neuronal cholinergic system components in neutrophils from COPD patients

**Fig. 1:**
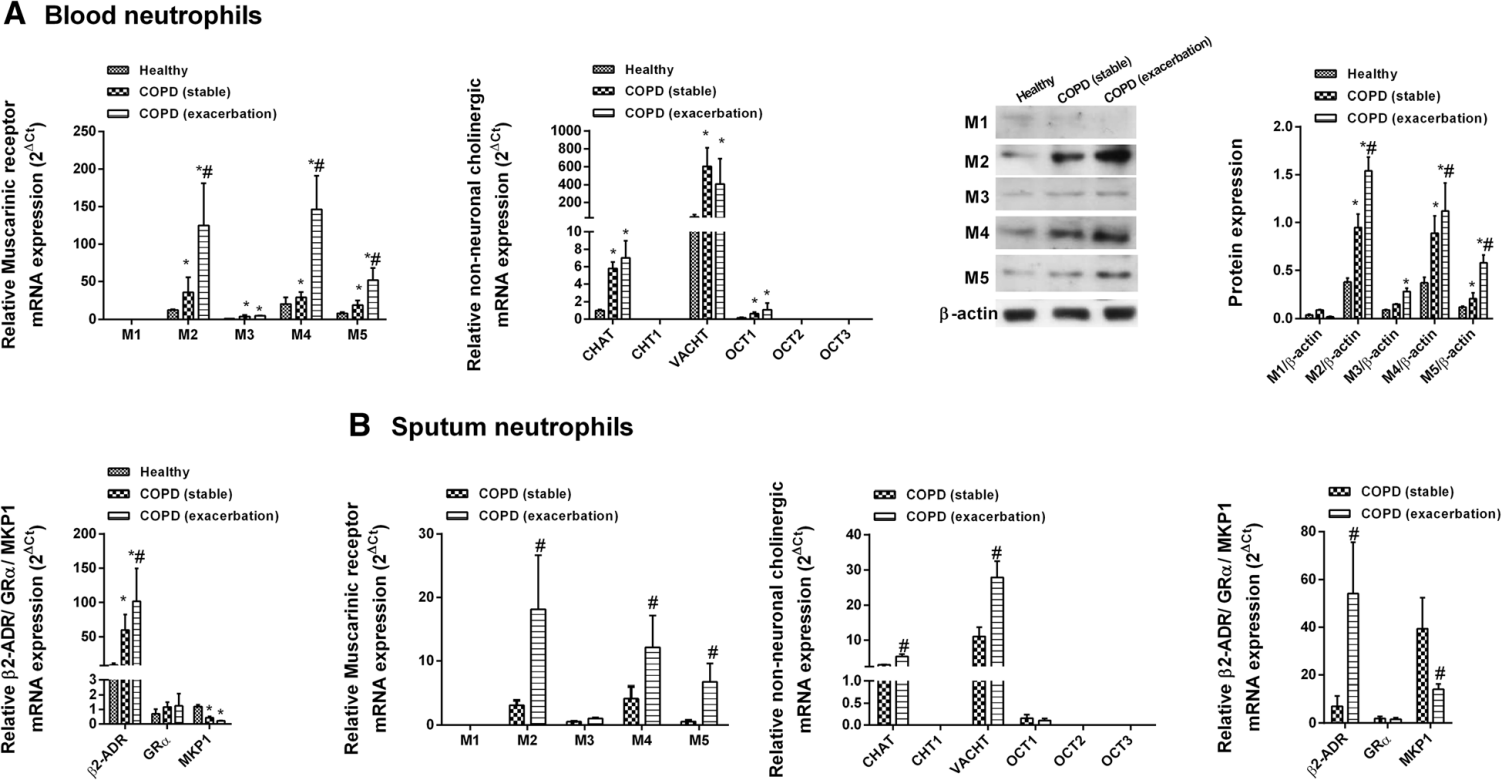
Expression of non-neuronal cholinergic system components in human neutrophils from healthy controls and COPD patients with stable and exacerbated disease. **a** Peripheral blood or **b** sputum neutrophils were isolated from healthy controls (*n* = 24) and patients with stable (*n* = 30) and exacerbated (*n* = 16) COPD. mRNA expression was measure by RT-PCR and quantified according to the 2^−ΔCt^ method, with expression of the housekeeping gene GAPDH serving as an internal control. Protein expression was measured by western blot and quantified based on the intensity of the target protein and the internal control β-actin. Representative images are shown. Data are presented as the mean ± SD. One-way repeated measures analysis of variance followed by post hoc Bonferroni tests: *p < 0.05 vs. healthy controls; #*p* < 0.05 vs. patients with stable COPD

Basal levels of the mRNA and protein of muscarinic receptor subtypes M2 and M4 were expressed in neutrophils from healthy individuals and from patients with stable COPD and induced in neutrophils from patients with exacerbated disease, in both peripheral blood and sputum (Fig. 1). M3 and M5 were detected at low levels but induced in neutrophils from patients with exacerbated COPD, while M1 expression was virtually absent. ChAT, the enzyme responsible for the generation of intracellular acetylcholine, was detected in blood and sputum neutrophils from healthy individuals and increased in COPD patients (Fig. 1). VAChT, responsible for loading acetylcholine into secretory organelles, was over-expressed in neutrophils from COPD patients (Fig. 1). OCT1, the transmembrane protein that transports acetylcholine, was detected at low levels but increased in COPD patients, while OCT2 and OCT3 were not detected (Fig. 1), nor was the high-affinity choline transporter ChT1. Beta 2 adrenoreceptor (β2-ADR) was detected in neutrophils from healthy donors and over-expressed in neutrophils from COPD patients, while there was no difference in GRα expression between groups. By contrast, MKP1 was down-regulated in neutrophils from patients with stable and exacerbated COPD (Fig. 1).

### Inhibition of neutrophil response by aclidinium bromide and fluticasone propionate

**Fig. 2:**
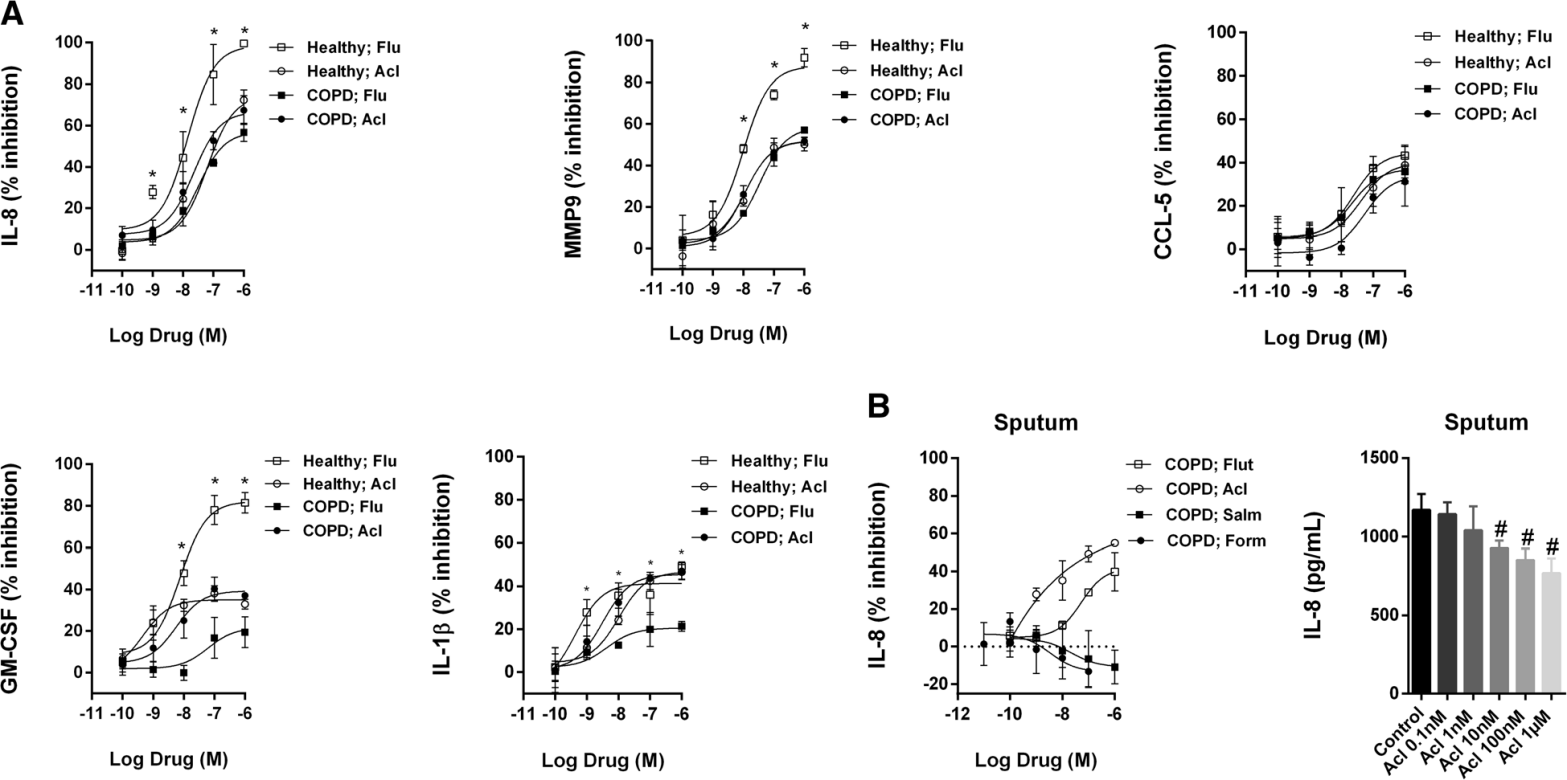
Aclidinium bromide shows anti-inflammatory properties in human neutrophils. Concentration-dependent inhibition of **a** lipopolysaccharide (LPS)- or **b** cigarette smoke extract (CSE)-induced cytokines or MMP-9 release by aclidinium bromide (Acl), fluticasone propionate (Flu), salmeterol (Salm) or formoterol (Form) from **a** peripheral blood and **b** sputum neutrophils of healthy controls and COPD patients. Neutrophils were preincubated with Acl (0.1 nM–1 μM), Flu (0.1 nM–1 μM), Salm (0.1 nM–1 μM) or Form (0.01–100 nM) for 1 h followed by cell stimulation with LPS (1 μg/ml) or CSE (5 %) for 6 h. The results are expressed as the mean ± SD (*n* = 4 each for cells from healthy controls and COPD patients in independent experiments with triplicate samples). A two-way ANOVA was followed by a post hoc Bonferroni test. *p < 0.05 vs. cells from COPD patients; #*p* < 0.05 vs. basal values

In peripheral blood neutrophils from healthy controls, fluticasone propionate (0.1 nM–1 μM) concentration-dependently inhibited the cytokine secretion induced by LPS (1 μg/ml), nearly completely suppressing IL-8, MMP9, GM-CSF and to a lesser extent IL-1β and CCL-5 secretion (Fig. 2a, Additional file 1: Table SE1). By contrast, the inhibitory effect of fluticasone propionate was impaired in neutrophils from COPD patients, with less than half of the percent maximum effect observed in healthy control neutrophils; the exception was CCL-5 (Fig. 2a, Additional file 1: Table SE1). Aclidinium bromide was less effective than fluticasone propionate in inhibiting cytokine secretion in neutrophils from healthy individuals, achieving 72, 50, 46, 39 and 33 % of the maximal inhibition for IL-8, MMP9, IL-1β, CCL-5 and GM-CSF, respectively; however, in neutrophils from COPD patients, it exhibited similar inhibitory effects. In sputum neutrophils from COPD patients stimulated with CSE 5 %, < 40 % of maximal IL-8 inhibition was achieved with fluticasone propionate (Fig. 2b, Additional file 1: Table SE1), whereas aclidinium bromide concentration-dependently inhibited CSE-induced IL-8 secretion in sputum neutrophils of COPD patients with similar potency and maximal effects observed in peripheral neutrophils from healthy and COPD patients. Aclidinium bromide also significantly inhibited basal IL-8 secretion in unstimulated sputum cells (Fig. 2b, Additional file 1: Table SE1). Formoterol exerted pro-inflammatory effects on IL-8, GM-CSF and IL-1β secretion in both peripheral blood and sputum neutrophils from healthy controls and COPD patients (Fig. 2b, Additional file 2: Figure SE1 and Additional file 1: Table SE1), while salmeterol showed weak inhibitory or pro-inflammatory effects depending on the measured cytokine (Fig. 2b, Additional file 2: Figure SE1 and Additional file 1: Table SE1). The pro-inflammatory actions of formoterol and salmeterol were inhibited by propranolol, which implied a role for β2-ADR (data not shown).

### Effect of the combination of aclidinium bromide and fluticasone propionate on neutrophil response

**Fig. 3:**
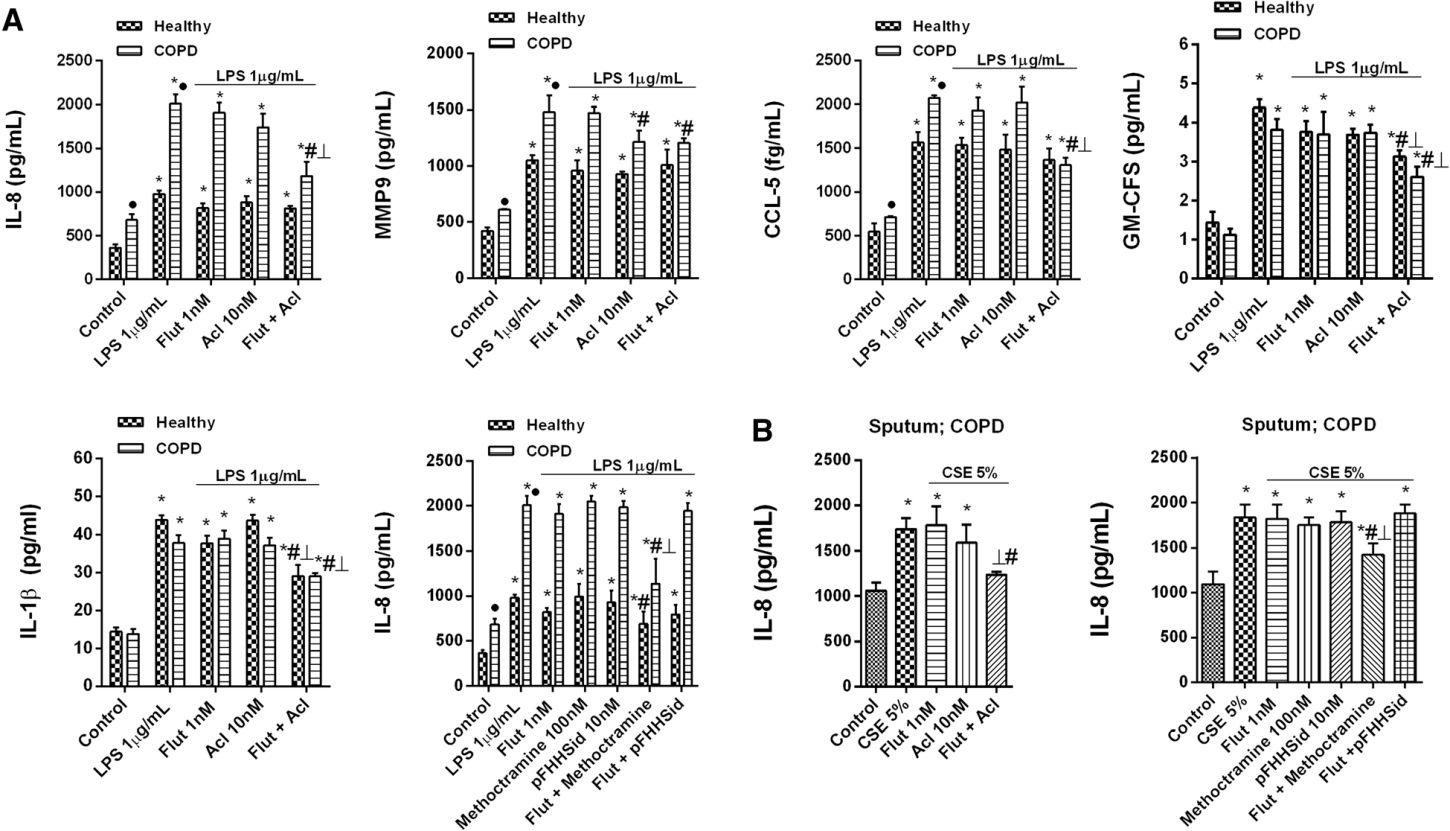
Effects of combined aclidinium bromide and fluticasone propionate on inflammatory cytokines in neutrophils from COPD patients. **a** Peripheral blood or **b** sputum neutrophils from healthy controls and COPD patients were incubated with aclidinium bromide (Acl), fluticasone propionate (Flut) or both for 1 h before they were stimulated with **a** lipopolysaccharide (LPS) or **b** cigarette smoke extract (CSE) for 6 h. Cytokine release was measured in cell supernatants. The results are expressed as the mean ± SD (*n* = 4 each for cells from healthy controls and COPD patients in independent experiments with triplicate samples). A two-way ANOVA was followed by a post hoc Bonferroni test. *p < 0.05 vs. control unstimulated cells; #*p* < 0.05 vs. stimulated cells. ⊥ *p* < 0.05 vs. monotherapy

Whether the anti-inflammatory effects of aclidinium bromide and fluticasone propionate are additive was tested using suboptimal concentrations of the drugs based on their concentration-dependent inhibitory curves. In peripheral blood neutrophils, the combination of fluticasone propionate (1 nM) and aclidinium bromide (10 nM) showed additive effects, resulting in nearly 50 % inhibition of LPS-induced IL-8, CCL-5, GM-CSF and IL-1β, but not MMP9 (Fig. 3). Suboptimal concentrations of fluticasone propionate in combination with 100 nM of the M2 inhibitor methoctramine [27] reproduced the additive effects observed with aclidinium bromide, whereas this was not the case with the M3 inhibitor pFHHSid (Fig. 3). Similar results were obtained in sputum neutrophils from COPD patients.

### Non-neuronal cholinergic activation in neutrophils from COPD patients

**Fig. 4:**
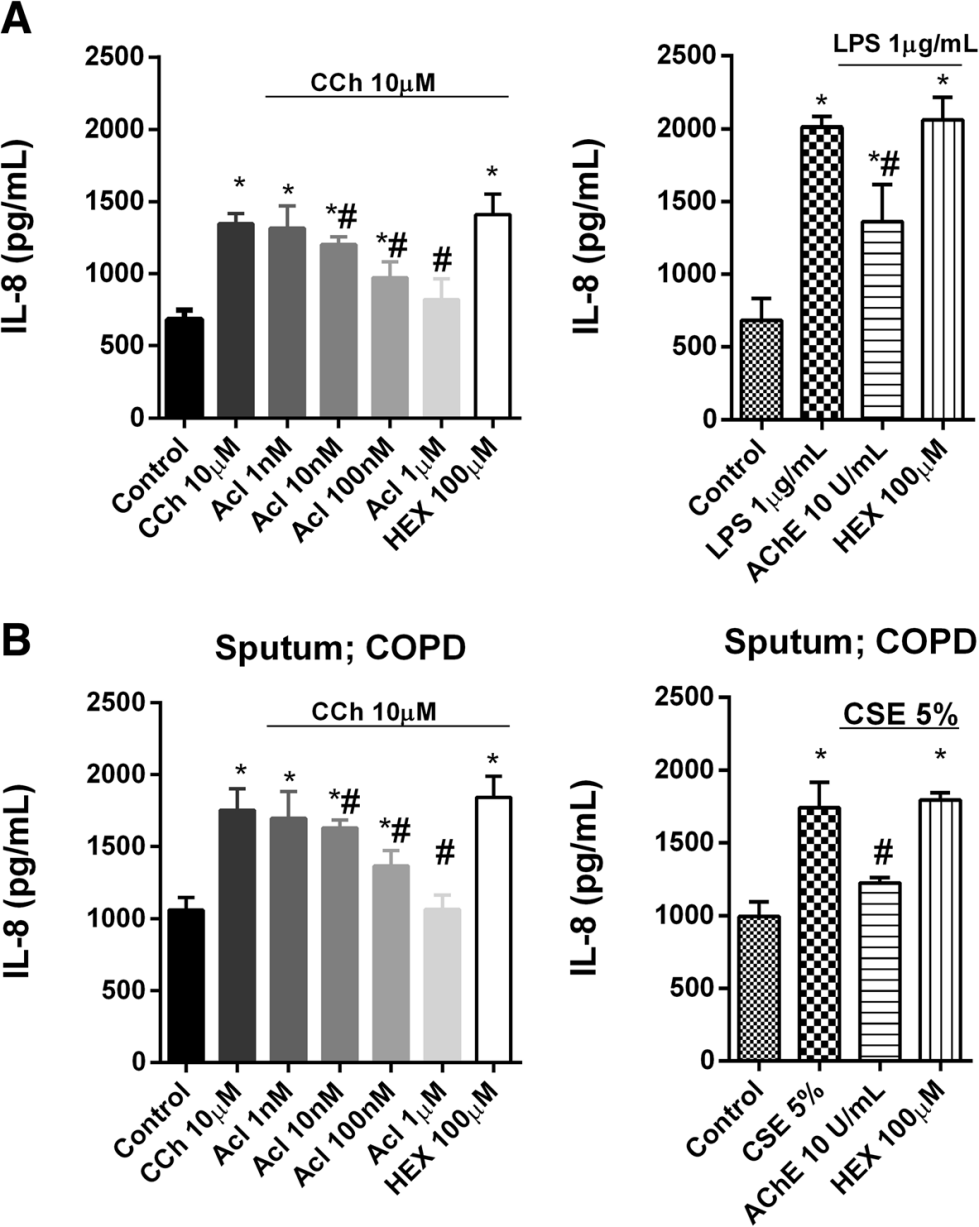
Activation of human neutrophils by cholinergic mediators. Neutrophils from the **a** peripheral blood or **b** sputum of COPD patients were incubated with aclidinium bromide (Acl), hexamethonium (HEX) or acetylcholinesterase (AChE) for 1 h and then stimulated with carbachol (CCh) or lipopolysaccharide (LPS) for 6 h. Cell supernatants were collected to measure interleukin (IL)-8 release. The results are expressed as the mean ± SD (*n* = 4 COPD cell populations in independent experiments run in triplicate). A two-way ANOVA was followed by a post hoc Bonferroni test. *p < 0.05 vs. control unstimulated cells; #*p* < 0.05 vs. stimulated cells

The muscarinic receptor agonist carbachol was used as a stimulus to explore functional muscarinic receptor activation in neutrophils. Carbachol (10 μM) induced IL-8 release in isolated blood and sputum neutrophils. This effect was concentration-dependently inhibited by aclidinium bromide but not by hexamethonium (Fig. 4), which ruled out the involvement of nicotinic receptors. To eliminate the contribution of extracellular acetylcholine to neutrophil activation, acetylcholinesterase was added 1 h before and again during neutrophil stimulation with LPS or CSE. Acetylcholinesterase attenuated the IL-8 release induced by LPS in peripheral blood neutrophils and by CSE in the sputum neutrophils of COPD patients, suggesting a role for extracellular acetylcholine in neutrophil activation (Fig. 4).

### Mechanisms involved in the improved anti-inflammatory effects of fluticasone propionate when combined with aclidinium bromide in neutrophils from COPD patients

**Fig. 5:**
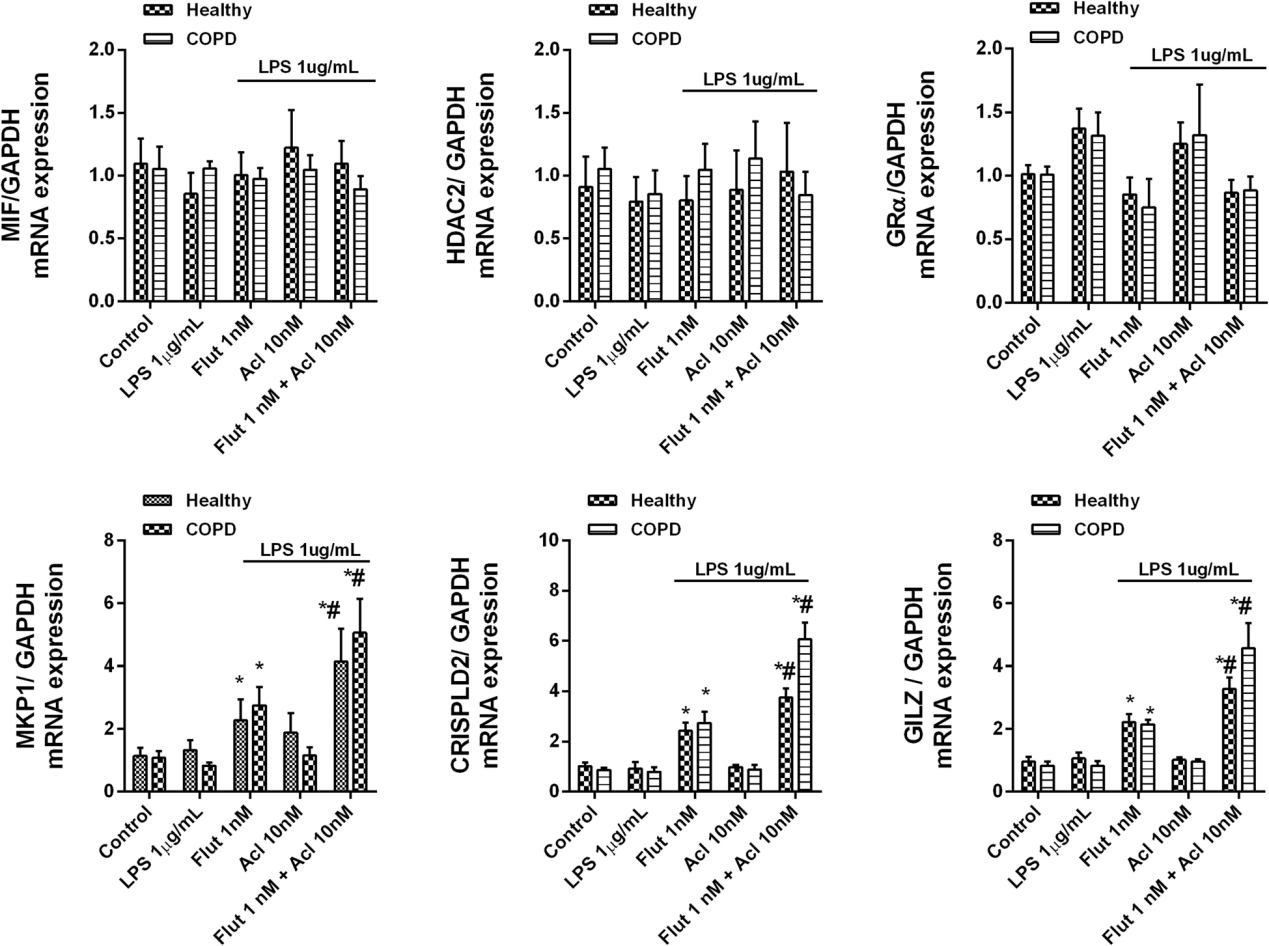
Effects of aclidinium bromide (Acl) and fluticasone propionate (Flut) on lipopolysaccharide (LPS)-induced corticosteroid modulators. Human peripheral blood neutrophils isolated from COPD patients were plated on 24-well plates, incubated with Acl, Flut or a combination thereof for 1 h, and stimulated with LPS for 6 h. Molecular corticosteroid modulators were quantified by RT-PCR using the 2^−ΔCt^ method, with expression of the housekeeping gene GAPDH serving as an internal control. The results are expressed as the mean ± SD (*n* = 4 each for cells from healthy controls and COPD patients in independent experiments with triplicate samples). A two-way ANOVA was followed by a post hoc Bonferroni test. *p < 0.05 vs. control unstimulated cells; #*p* < 0.05 vs. Flut monotherapy

In peripheral blood neutrophils from COPD patients, the combination of fluticasone propionate and aclidinium bromide did not affect the expression of corticosteroid modulator molecules including macrophage migration inhibitory factor (MIF), HDAC2 and GRα. However, together the two drugs increased the expression of the corticosteroid-inducible genes MKP1, CRISPLD2 and GILZ to a greater extent than achieved with fluticasone propionate monotherapy (Fig. 5), consistent with the increased activation of the glucocorticoid response element (GRE).

**Fig. 6:**
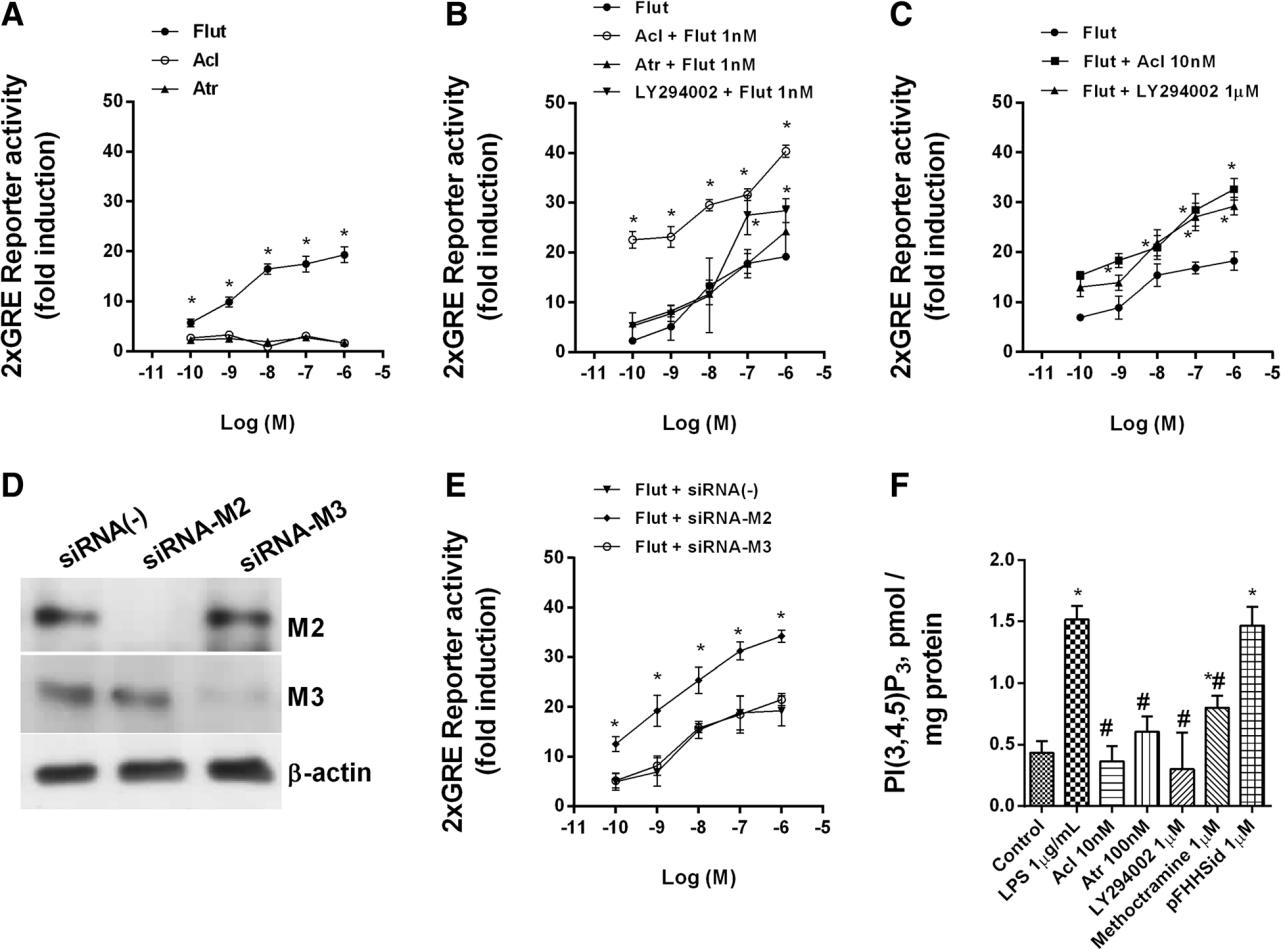
Effects of aclidinium bromide on glucocorticoid response element (GRE) signal and phosphatidylinositol-3-kinase delta activation (PI3Kδ). **a**–**d** Bronchial epithelial Beas2B cells were transfected with a GRE reporter gene and stimulated with different combinations of aclidinium bromide (Acl), atropine (Atr), fluticasone propionate (Flut) and the inhibitor of PI3K LY294002. **d** Beas2b transfected with silencing (siRNA) negative control, and siRNA for muscarinic receptors M2 and M3 receptors were verified by western blot analysis. **e** Beas2b cells transfected with the GRE reporter gene and subjected to siRNA of the genes encoding muscarinic receptors M2 and M3 were stimulated with Flut. The results are expressed as the mean ± SD of three independent experiments run in triplicate. One-way ANOVA was followed by a post hoc Bonferroni test. *p < 0.05 vs. Flut monotherapy. **f** Peripheral blood neutrophils from COPD patients were incubated with Acl for 1 h and stimulated with lipopolysaccharide (LPS) for 30 min. PI3Kδ activity in human neutrophils from COPD patients. The results are expressed as the mean ± SD (*n* = 4 COPD cell populations in independent experiments run in triplicate). A two-way ANOVA was followed by a post hoc Bonferroni test. *p < 0.05 vs. control unstimulated cells; #*p* < 0.05 vs. LPS-stimulated cells

**Fig. 7:**
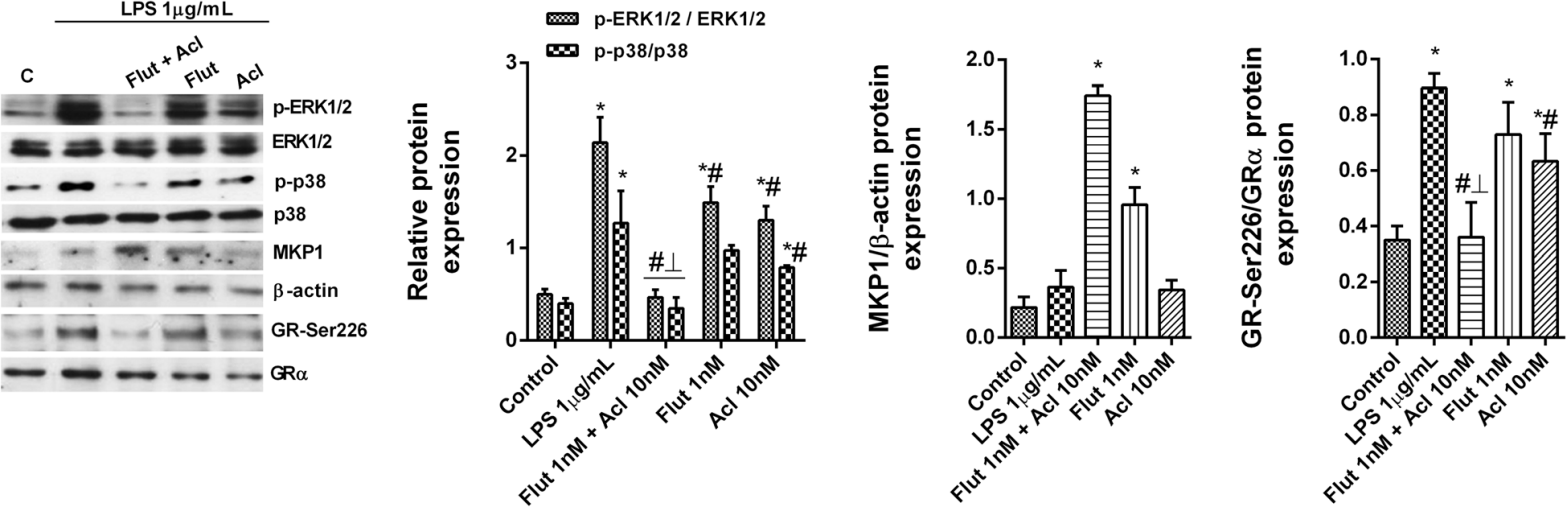
Combined aclidinium bromide (Acl) and fluticasone propionate (Flut) shows additive effects in inhibiting the lipopolysaccharide (LPS)-induced phosphorylation of ERK1/2, p38 and GR-Ser226 and in increasing MKP1 levels. Human peripheral blood neutrophils from COPD patients were incubated with Acl, Flut or a combination thereof for 1 h and then stimulated with LPS during 30 min. Total protein was extracted for western blotting. The expression of p-ERK1/2, p-p38 and p-GR-Ser226 was determined as the ratio of the respective non-phosphorylated forms, and that of MKP1 vs. β-actin expression. Representative images are shown. Data are presented as the mean ± SD (*n* = 4 COPD cell populations in independent experiments run in triplicate). A two-way repeated measures ANOVA was followed by post hoc Bonferroni tests: *p < 0.05 vs. the control; #*p* < 0.05 vs. LPS-stimulated cells. ⊥ *p* < 0.05 vs. cells treated with drug monotherapy

To corroborate this hypothesis, bronchial epithelial cells Beas2B were transfected with a GRE reporter construct. Treatment of these 2xGRE-Beas2B reporter cells with dexamethasone (0.1 nM–1 μM) for 6 h induced GRE-dependent transcription in a concentration-dependent manner (Fig. 6a). Neither aclidinium bromide nor atropine as monotherapy modified the GRE signal (Fig. 6a), whereas it was increased significantly by combinations of increasing concentrations of aclidinium bromide, atropine or the PI3K inhibitor LY294002 with a fixed (1 nM) concentration of fluticasone propionate (Fig. 6b). Similar results were obtained with combinations of fixed concentrations of aclidinium bromide (10 nM) or LY294002 (1 μM) with increasing concentrations of fluticasone propionate (Fig. 6c). In siRNA-M2 cells, fluticasone-propionate-induced GRE activation was higher than in siRNA-M3 or control siRNA(−) cells, suggesting the participation of M2 receptors (Fig. 6d). Aclidinium bromide, atropine, methoctramine and LY294002 (but not pFHHSid) inhibited LPS-induced PI3Kδ activity in COPD blood neutrophils (Fig. 6e). Additional mechanistic investigations showed that aclidinium bromide in combination with fluticasone propionate potentiated the inhibition of p-ERK1/2, p-p38 and GRser226 and increased the expression of MKP1 over the level achieved with either drug alone (Fig. 7).

## Discussion

This study provided novel evidence of the additive anti-inflammatory properties of aclidinium bromide and fluticasone propionate in neutrophils from COPD patients. It thus establishes a scientific rationale for future clinical research with ICS/LAMA combinations in COPD. We also demonstrated activation of the non-neuronal cholinergic system in the blood and sputum neutrophils of COPD patients and the effective reduction of cytokine and metalloproteinase release in aclidinium-bromide-treated neutrophils from COPD patients. The combination of aclidinium bromide and fluticasone propionate increased the impaired anti-inflammatory properties of the latter drug by a mechanism involving the inhibition of GRα phosphorylation at Ser-226, the enhancement of fluticasone-propionate-mediated GRE activation and the expression of corticosteroid-dependent anti-inflammatory genes including MKP1, CRISPLD2 and GILZ.

In the treatment of mild to severe COPD, ICS in combination with LABAs plays an essential role in patients at risk of disease exacerbation. Although recent randomised clinical trials showed that ICS withdrawal did not increase the number of exacerbations in patients with severe COPD under LABA/LAMA/ICS triple therapy [28], the reduced FEV1 and impaired quality of life after ICS cessation provided evidence of the benefit of ICS in COPD [28, 29]. However, ICS monotherapy is not indicated; instead, the combination of ICS with LABAs has been broadly prescribed as it is more effective than either agent alone in improving lung function and health status and in reducing exacerbation in patients with moderate to very severe COPD [2]. Nonetheless, the recommendation for combined ICS/LAMA is not evidence-based.

The presence of a non-neuronal cholinergic system in the alveolar macrophages and neutrophils of COPD patients has been implicated in the pathogenesis of the disease [15, 16]. It was reported that activation of M1, M2 and M3 receptors occurred in the sputum neutrophils of COPD patients by the addition of exogenous acetylcholine [16]. Immunocytochemistry demonstrated the reduced expression of M2 in COPD compared with that in healthy neutrophils and an increase in M3 expression [16]. In the present work, by fully characterising the non-neuronal cholinergic system in blood and sputum neutrophils from healthy controls and in COPD patients, we found that M2 was more highly expressed in patients with stable disease and over-expressed in those with exacerbated disease. Similar results were obtained for M4, to a lesser extent for M5 and to a very slight extent for M3. The discrepancy with the previous report regarding M2 expression might be explained by the different techniques and antibodies used. However, we also measured expression by RT-PCR and western blot and obtained similar results. In neutrophils from patients with stable or exacerbated COPD, the non-neuronal cholinergic system was over-expressed, with a predominance of M2 and M4 receptors as well as ChAT, VACHT and OCT1. These findings suggest that human COPD neutrophils synthesise intracellular acetylcholine, mediated by ChAT, which is loaded into secretory organelles by VAChT, thereby making acetylcholine available for secretion through OCT1 membrane channels. Carbachol, a stable analogue of acetylcholine, activated neutrophils to release IL-8, whereas inhibition of the latter by aclidinium bromide seemed to confirm a role for functional muscarinic receptors. The addition of exogenous acetylcholinesterase to eliminate acetylcholine from the culture medium reduced neutrophil activation by LPS in blood neutrophils and by CSE in sputum neutrophils, suggesting that bacterial infection and cigarette smoke activate acetylcholine release from neutrophils, which in turn promotes the release of the cytokines and metalloproteinases induced by these triggers. However, we did not detect acetylcholine in the culture medium of human neutrophils, probably due to the low sensitivity of the commercial kit used in this study or rapid degradation by acetylcholinesterases [30] (data not shown). Similar results have been obtained in alveolar macrophages stimulated with carbachol, in which the release of leukotriene B4 via M3 receptor activation was described [15]. In this work, the inhibitory effect of aclidinium bromide appeared to be mediated by M2 blockade, since the M2 antagonist methoctramine inhibited cytokine release but pFHHSid, an antagonist of M3, did not.

Neutrophils isolated from our COPD patients were less sensitive to the anti-inflammatory effects of fluticasone propionate than neutrophils from healthy controls, as previously reported in a study in which dexamethasone was the corticosteroid [10]. Fluticasone propionate in combination with aclidinium bromide exhibited additive anti-inflammatory effects in the blood and sputum neutrophils of COPD patients, consistent with the increased anti-inflammatory effects of budesonide combined with the anti-muscarinic R,R-glycopyrrolate in LPS-stimulated human monocytes [31]. The additive effects achieved with aclidinium bromide and fluticasone propionate can be attributed to M2 receptor antagonism, since methoctramine, but not pFHHSid, increased the anti-inflammatory effects of fluticasone propionate. However, the involvement of M4 and M5 cannot be ruled out by our data.

M1, M3 and M5 receptors are coupled to the G_q_ protein and mediate bronchial contraction by activating phospholipase Cβ1 (PLC), which leads to the production of inositol 1,4,5-trisphosphate (IP_3_); the latter is necessary to activate the release of intracellular calcium stores. M2 and M4 receptors are coupled to G_i_ protein and mediate PI3K activation and the inhibition of adenylate cyclase and cyclic adenosine monophosphate (cAMP) [17]. In this work, both aclidinium bromide and methoctramine suppressed LPS-induced PI3Kδ activity. PI3Kδ is increased in neutrophils [10] and macrophages [32] from COPD patients and mediates their corticosteroid insensitivity. Therefore, inhibition of PI3Kδ activity by aclidinium bromide through the M2 receptor may at least partially explain the improved effects achieved with the further addition of fluticasone propionate. cAMP also increases the anti-inflammatory effects of corticosteroids. Although not explored in this work, we previously showed that, in human fibroblasts, aclidinium bromide prevents the down-regulation of cAMP induced by carbachol activation [14], which would also account for the additive effect of aclidinium bromide in combination with fluticasone propionate. Further evidence is provided by the fact that cAMP inducers such as LABAs and roflumilast enhance the effects of corticosteroids by elevating cAMP [10, 33].

The anti-inflammatory effect of corticosteroids is mediated in part by promoting the nuclear translocation of GRα to nuclear GRE regions, which in turn increases the expression of anti-inflammatory genes. In this work, we demonstrated an increased fluticasone-propionate-mediated GRE signal by the further addition of aclidinium bromide, which resulted in additive effects on the stimulation of corticosteroid-inducible genes such as MKP-1, CRISPLD2 and GILZ. MKP1 dephosphorylates and inactivates different mitogen-activated kinases such as ERK1/2 and p38 as part of the anti-inflammatory effects of corticosteroids. Recent evidence indicated that the inhibition of GRα via GR-Ser-226 phosphorylation by p38, ERK1/2 or JNK1 inhibits GRα nuclear translocation and thus mediates corticosteroid insensitivity in asthmatics [34, 35]. Accordingly, the additive effects obtained with the combination of aclidinium bromide and fluticasone propionate in increasing MKP1 and inhibiting p-ERK1/2 and p-p38 could explain the reduced expression of GR-Ser-226 phosphorylation and therefore the increased anti-inflammatory effects of the drug combination. CRISPLD2 is a secreted protein that binds LPS in humans. Enhanced CRISPLD2 expression by fluticasone propionate down-regulates LPS-activating toll-like receptor 4 (TLR4) pro-inflammatory responses, thus perhaps reducing the exacerbations of COPD produced by infections with gram-negative bacteria [36]. In this work, LPS was used as the stimulus and its pro-inflammatory effects were enhanced by cigarette smoke, mediated in part via TLR4 activation [37]. Thus, the inhibition of TLR4 downstream signalling in response to the increase in CRISPLD2 induced by the combination of aclidinium bromide and fluticasone propionate may explain at least some of the anti-inflammatory effects of the two drugs. The corticosteroid-inducible gene GILZ inhibits the transcriptional activity of NF-kB and AP-1, both of which are involved in inflammatory pathways [38]. By suppressing indices of inflammation, the increase in GILZ expression and that of other corticosteroid-inducible genes by the drug combination could confer protection against bronchoconstriction, thus limiting airway remodelling. However, the present work was confined to an in vitro analysis of the potential clinical benefits of LAMA/ICS combinations and its results still require clinical corroboration.

## Conclusions

Aclidinium bromide attenuates corticosteroid-resistant neutrophil activation in COPD patients. Additive anti-inflammatory properties are achieved when it is used in combination with fluticasone propionate. These observations provide a scientific rationale for treating COPD patients with a combination of LAMAs and corticosteroids.

## Additional files

Additional file 1: Supplementary Table S1. Maximal percentage of inhibition of IL-8, MMP-9, CCL-5, GM-CSF and IL-1β release from neutrophils of healthy subjects and COPD patients. Neutrophils were incubated with aclidinium (Acl; 0.1nM-1 μM), fluticasone (Flut; 0.1nM-1 μM), formoterol (Form; 0.01nM-100nM) or salmeterol (Salm; 0.1nM-1 μM) in response to LPS (1 μg/ml) or cigarette smoke extract (CSE 5 %). The levels of different cytokines in the cell supernatant were determined and the maximal percent of inhibitions were calculated. Values are mean ± SD of 3 independent experiments run in triplicate. **p* < 0.05 vs Healthy values; #*p* < 0.05 vs Acl group. (DOCX 17 kb)
Additional file 2: Supplementary Figure E1. Effects of formoterol and salmeterol on pro-inflammatory markers. Concentration-dependent inhibition of lipopolysaccharide (LPS)-induced cytokines or MMP-9 release by formoterol (Form) and salmeterol (Salm) from peripheral blood neutrophils of healthy subjects and COPD patients. Neutrophils were preincubated with Salm (0.1nM-1 μM) or form (0.01nM-100nM) for 1 h followed by cell stimulation with LPS (1 μg/ml) for 6 h. Results are expressed as means ± SD of *n* = 3 (3 cell healthy and 3 cell COPD populations run in triplicate) independent experiments. Two-way ANOVA was followed by the post hoc Bonferroni test. **p* < 0.05 related to cells from COPD patients; #*p* < 0.05 related to the basal values. (TIF 3172 kb)

## Abbreviations

AChE: Acetylcholinesterase
COPD: Chronic obstructive pulmonary disease
CRISPLD2: Cysteine-rich secretory protein LCCL domain-containing 2
CSE: Cigarette smoke extract
FEV1: Forced expiratory volume in one second
FVC: Forced vital capacity
GILZ: Glucocorticoid-induced leucine zipper
GOLD: Global Initiative for Chronic Obstructive Lung Disease
GRE: Glucocorticoid response element
GRα: Glucocorticoid receptor alpha
HDAC2: Histone deacetylase-2
ICS: Inhaled corticosteroid
LABA: Long-acting beta adrenergic agonist
LAMA: Long-acting muscarinic antagonist
LPS: Lipopolysaccharide
MIF: Macrophage migration inhibitory factor
MKP-1: Mitogen-activated protein kinase phosphatase 1
PDE4: Phosphodiesterase-4
PI3Kδ: Phosphoinositide-3-kinase delta
VAChT: Vesicular acetylcholine transporter
β2ADR: Beta-2 adrenergic receptor
