# Supplementary material for: Non-neuronal cholinergic system contributes to corticosteroid resistance in chronic obstructive pulmonary disease patients
Source: Respir Res. 2016 Nov 8;17:145. doi: 10.1186/s12931-016-0467-8 (PMC5101693; doi:10.1186/s12931-016-0467-8)
Supplement: Additional file 1: — Supplementary Table S1. Maximal percentage of inhibition of IL-8, MMP-9, CCL-5, GM-CSF and IL-1β release from neutrophils of healthy subjects and COPD patients. Neutrophils were incubated with aclidinium (Acl; 0.1nM-1 μM), fluticasone (Flut; 0.1nM-1 μM), formoterol (Form; 0.01nM-100nM) or salmeterol (Salm; 0.1nM-1 μM) in response to LPS (1 μg/ml) or cigarette smoke extract (CSE 5 %). The levels of different cytokines in the cell supernatant were determined and the maximal percent of inhibitions were calculated. Values are mean ± SD of 3 independent experiments run in triplicate. *p < 0.05 vs Healthy values; #p < 0.05 vs Acl group. (DOCX 17 kb) [file 12931_2016_467_MOESM1_ESM.docx]

**Supplementary table S1.** Maximal percentage of inhibition of IL-8, MMP-9, CCL-5, GM-CSF and IL-1β release from neutrophils of healthy subjects and COPD patients. Neutrophils were incubated with aclidinium (Acl; 0.1nM-1µM), fluticasone (Flut; 0.1nM-1µM), formoterol (Form; 0.01nM-100nM) or salmeterol (Salm; 0.1nM-1μM) in response to LPS (1µg/ml) or cigarette smoke extract (CSE 5%). The levels of different cytokines in the cell supernatant were determined and the maximal % of inhibitions were calculated. Values are mean ± SD of 3 independent experiments run in triplicate. *p < 0.05 vs Healthy values; #p<0.05 vs Acl group.

|  | Stimulus | Treatment | Maximal % inhibition (blood neutrophils) | | Maximal % inhibition (sputum neutrophils) |
| --- | --- | --- | --- | --- | --- |
|  |  |  | Healthy | COPD | COPD |
| IL-8 | LPS | Acl | 72.4 ± 8.4 | 67.4 ± 12.1 |  |
|  |  | Flut | 99.5 ± 1.9# | 56.7 ± 7.6*# |  |
|  |  | Form | -112.6 ± 23.3# | -51.9 ± 30.5*# |  |
|  |  | Salm | 46.7 ± 3.4# | -35.5 ± 10.6*# |  |
| MMP-9 | LPS | Acl | 50.2 ± 5.4 | 51.6 ± 3.35 |  |
|  |  | Flut | 91.8 ± 7.7# | 56.9 ± 9.9* |  |
|  |  | Form | 4.6 ± 9.9# | 23.7 ± 21* |  |
|  |  | Salm | 48.2 ± 7 | 21.15 ± 9.9* |  |
| CCL-5 | LPS | Acl | 38.8 ± 14.8 | 31.15 ± 19.5 |  |
|  |  | Flut | 43.1 ± 8.1 | 35.8 ± 12.1 |  |
|  |  | Form | 37.5 ± 18.4# | 27.5 ± 5 |  |
|  |  | Salm | 20 ± 5.3 | 29.3 ± 9.4 |  |
| GM-CSF | LPS | Acl | 32.8 ± 4.8 | 37.1 ± 2.47 |  |
|  |  | Flut | 81.4 ± 20.4# | 19.4 ± 12.8*# |  |
|  |  | Form | -26 ± 16.8 | -6.5 ± 15.32 |  |
|  |  | Salm | 16 ± 15.5 | 1.24 ± 12.3 |  |
| IL-1β | LPS | Acl | 46.6 ± 5.8 | 47.3 ± 6.9 |  |
|  |  | Flut | 49.3 ± 3.3# | 21.3 ± 4*# |  |
|  |  | Form | -24.1 ± 15.4# | 39.9 ± 10.5* |  |
|  |  | Salm | 16.2 ± 12 | 11.5 ± 1.9* |  |
| IL-8 | CSE | Acl |  |  | 54.9 ± 1.9 |
|  |  | Flut |  |  | 39.7 ± 17.5# |
|  |  | Form |  |  | -13.2 ± 14.11# |
|  |  | Salm |  |  | -10.8 ± 15.6# |
